# Supplementary material for: Current Status and Management of Chronic Myeloid Leukemia in the Gulf Region: Survey Results and Expert Opinion
Source: Cancers (Basel). 2024 May 31;16(11):2114. doi: 10.3390/cancers16112114 (PMC11172167; doi:10.3390/cancers16112114)

CML Treatment

- **For how long have you been treating CML?**

(Number of respondents=13)

a) Less than 2 years
b) Between 2 and 5 years
c) Between 5 and 10 years
d) Between 10 and 15 years
e) More than 15 years

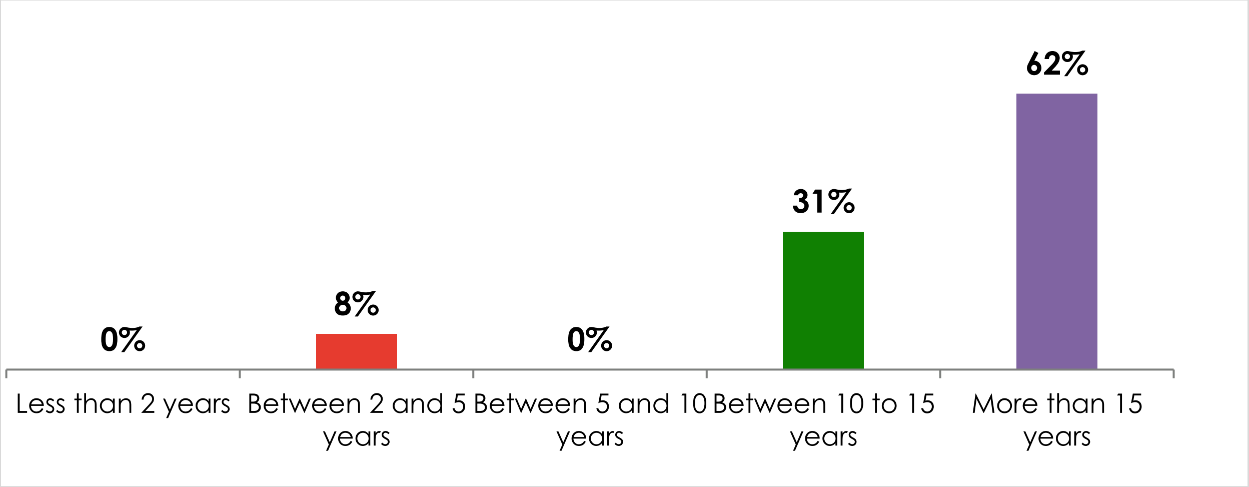

- **To how many CML patients have you personally provided care in the previous 12 months, including patients you are no longer treating?**

(Number of respondents=13)

a) Less than 5 years
b) Between 5 and 10
c) Between 10 and 20
d) Between 20 and 50
e) Between 50 and 100
f) More than 100

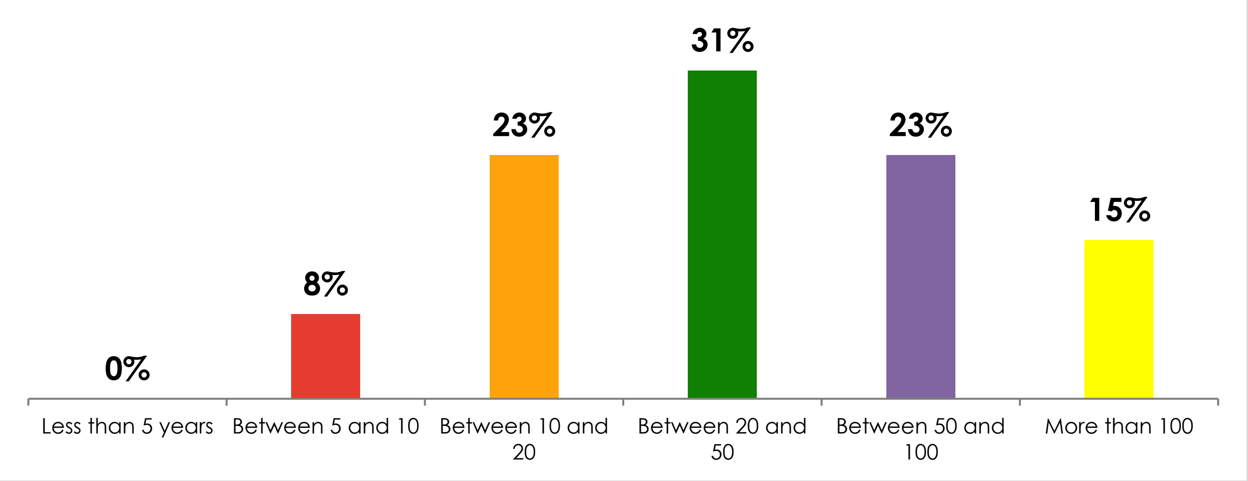

- **Currently, what % of your CML patients are in each line of therapy?**

(Number of respondents=13)

a) First Line
b) Second Line
c) Third Line

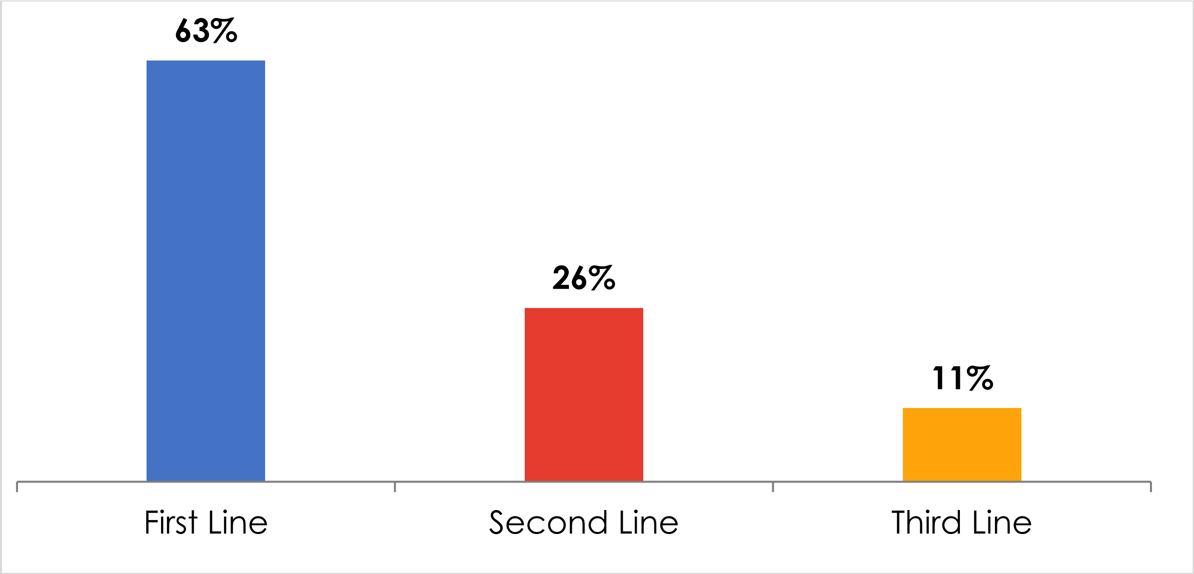

<u>CML Diagnosis</u>

- **When you have a patient with a clinical presentation compatible with CML diagnosis, which initial tests do you perform?**

(Number of respondents=13)

a) Karyotyping
b) Quantitative PCR
c) Qualitative PCR
d) FISH
e) Mutation analysis

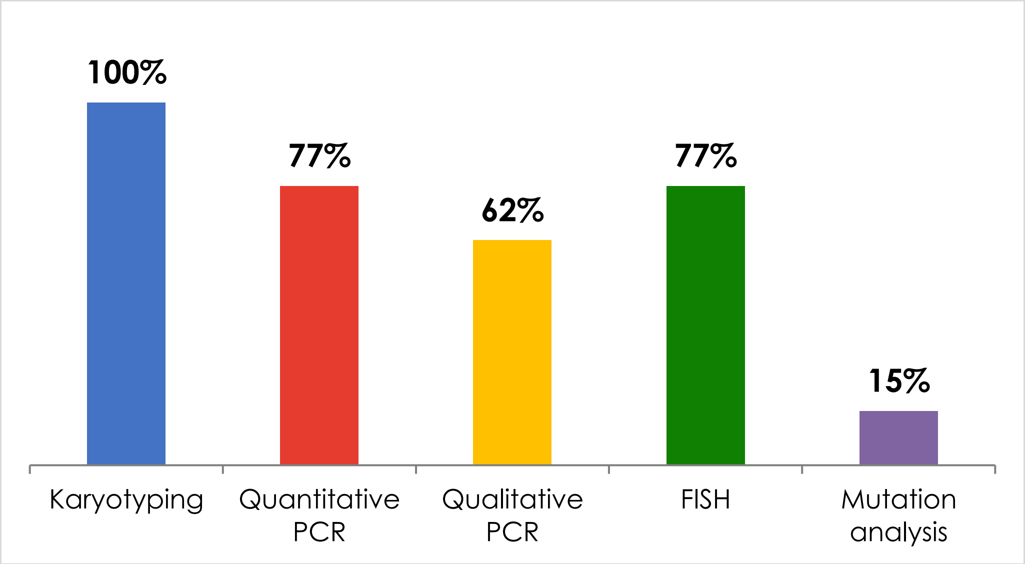

- **If karyotyping is performed, how is it collected?**

(Number of respondents=13)

a) Bone marrow
b) Blood sample

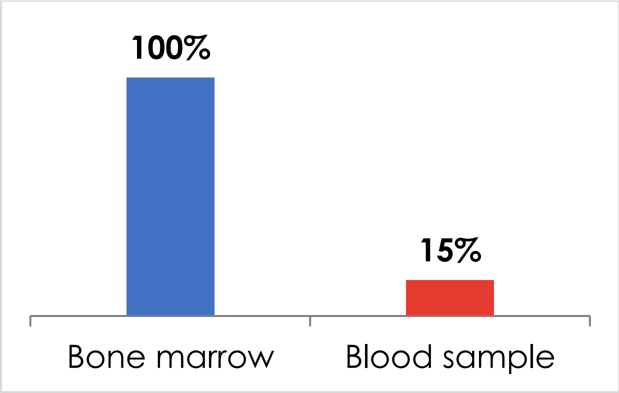

Conversion factor & standardization process

- **How is the conversion factor established in your center?**

(Number of respondents=13)

a) International reference lab
b) National reference lab
c) Commercial standards
d) Don't know
e) Not established yet

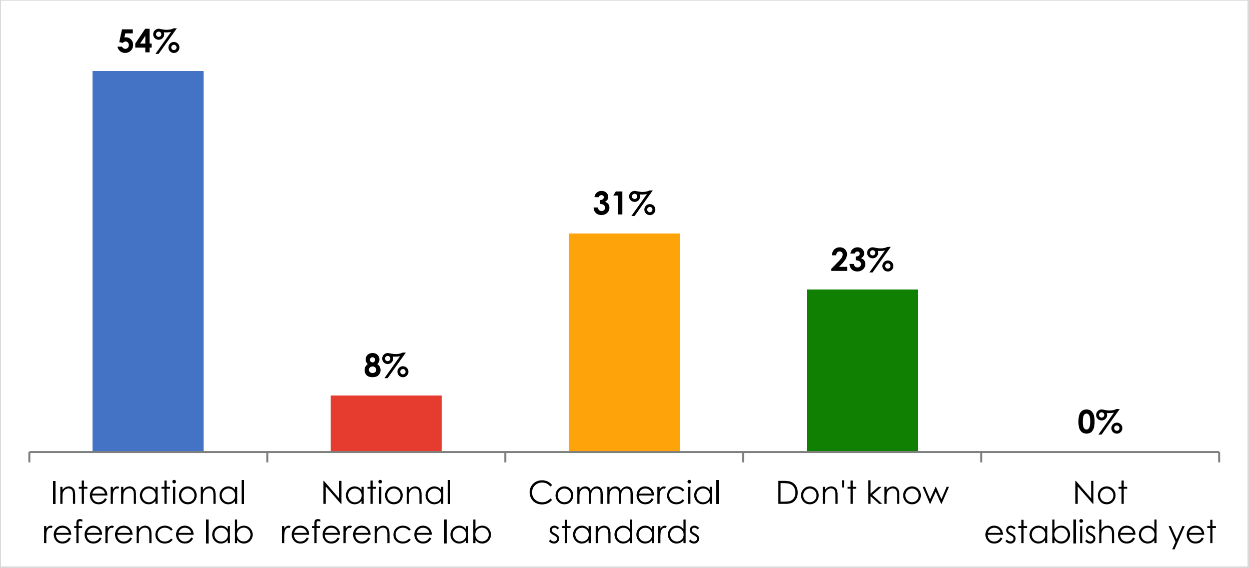

- **When was the last standardization process done?**

(Number of respondents=13)

a) Less than 6 months ago
b) Between 6 and 12 months
c) Don't know

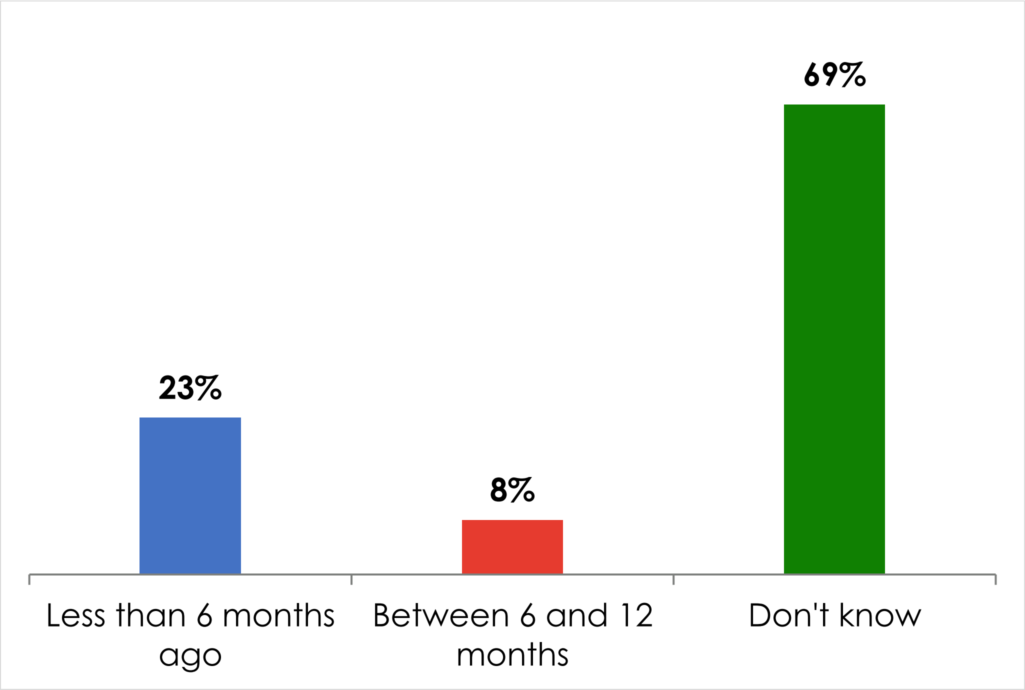

- **What level is the Quantitative real-time PCR available at your center able to detect (limit of detection)?**

(Number of respondents=10)

a) MR4
b) MR4.5
c) MR5
d) Other

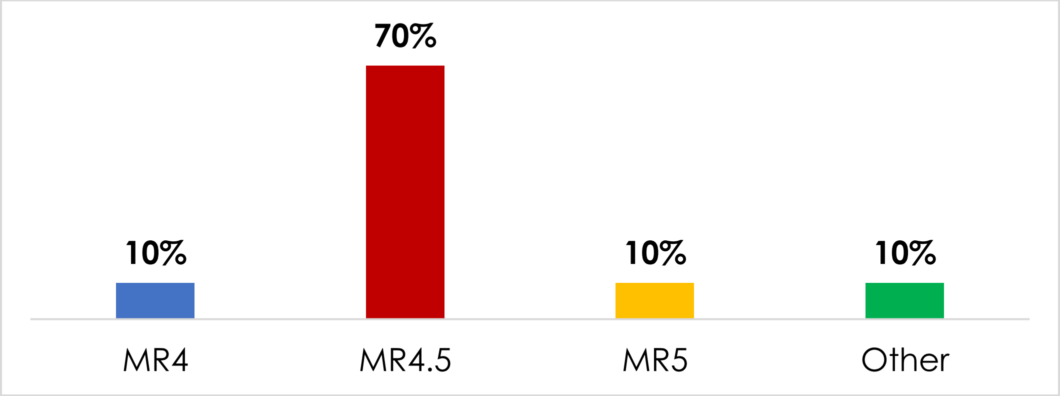

*Other = 4.96

- **Do you have access to deep molecular remission testing by Quantitative real-time PCR to a level of MR4 or deeper?**

(Number of respondents=10)

a) Yes
b) No

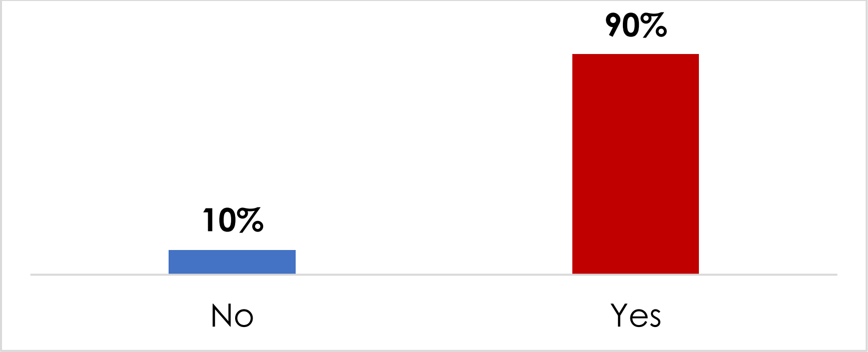

- **When you receive a Quantitative real-time PCR lab report with BCR-ABL level as "not detectable", you would be confident that the response is:**

(Number of respondents=10)

a) MMR
b) MR4
c) MR4.5 and above

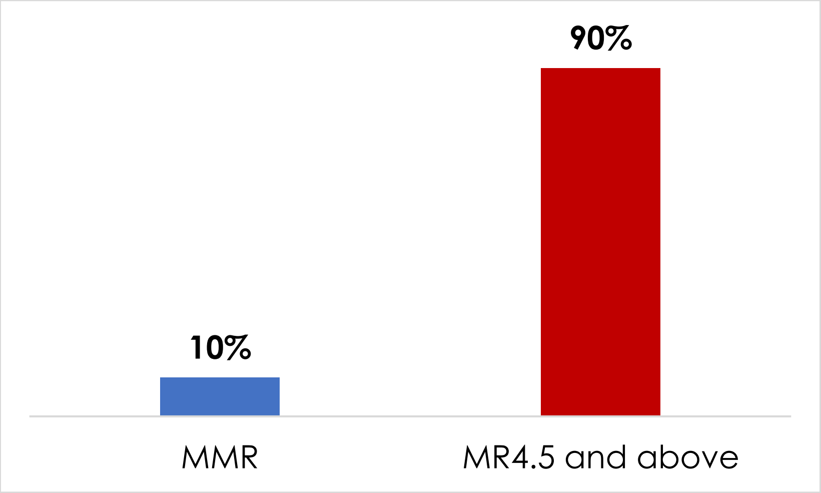

- **Which of the following information is provided in the BCR-ABL lab report?**

(Number of respondents=10)

a) Assay type
b) Results in IS
c) BCR-ABL copy number
d) Control gene copy number
e) Trends over time
f) Raw results

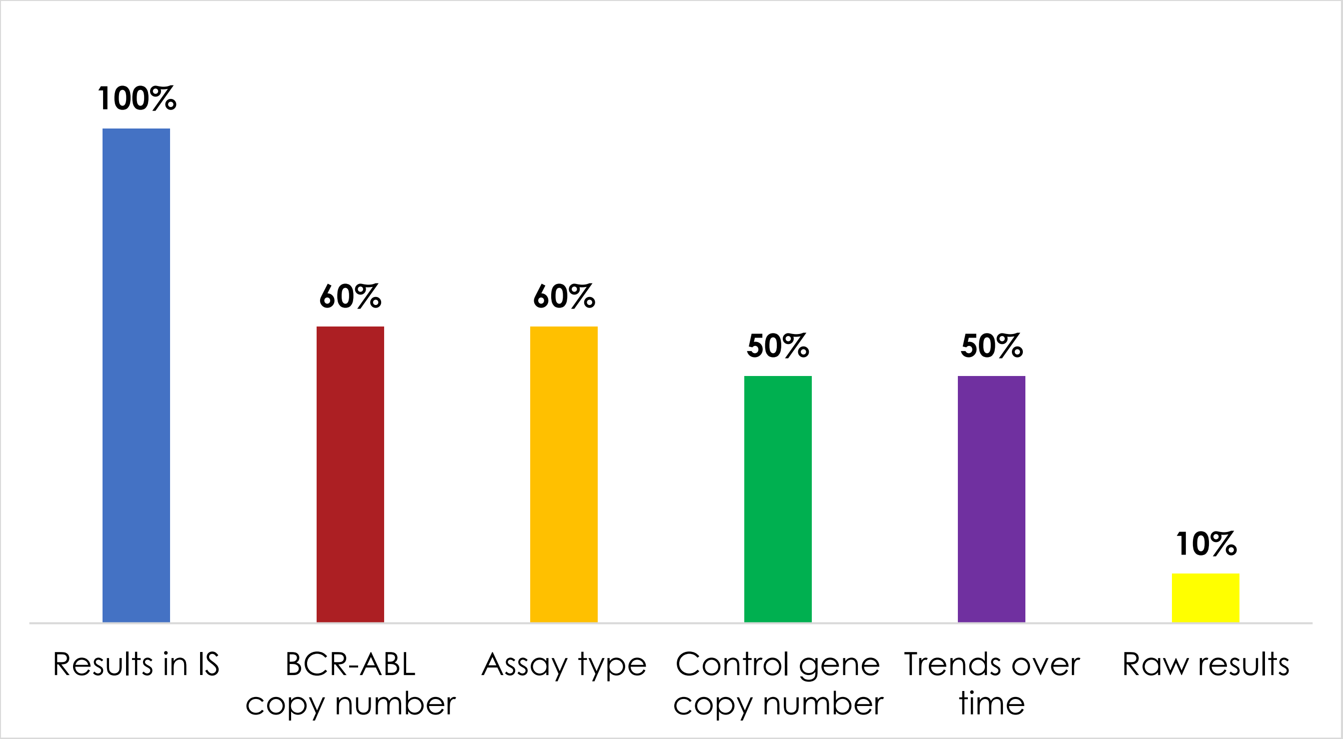

- **When do you assess mutation status?**

(Number of respondents=10)

a) Treatment failure excluding intolerance
b) Loss of MMR while on treatment
c) Progression to accelerated or blast phase
d) Any sign of loss of response (hematologic or cytogenetic)
e) Significant BCR-ABL rise, from 0.001 to 0.1 (1 log increase) after achieving MMR
f) Never

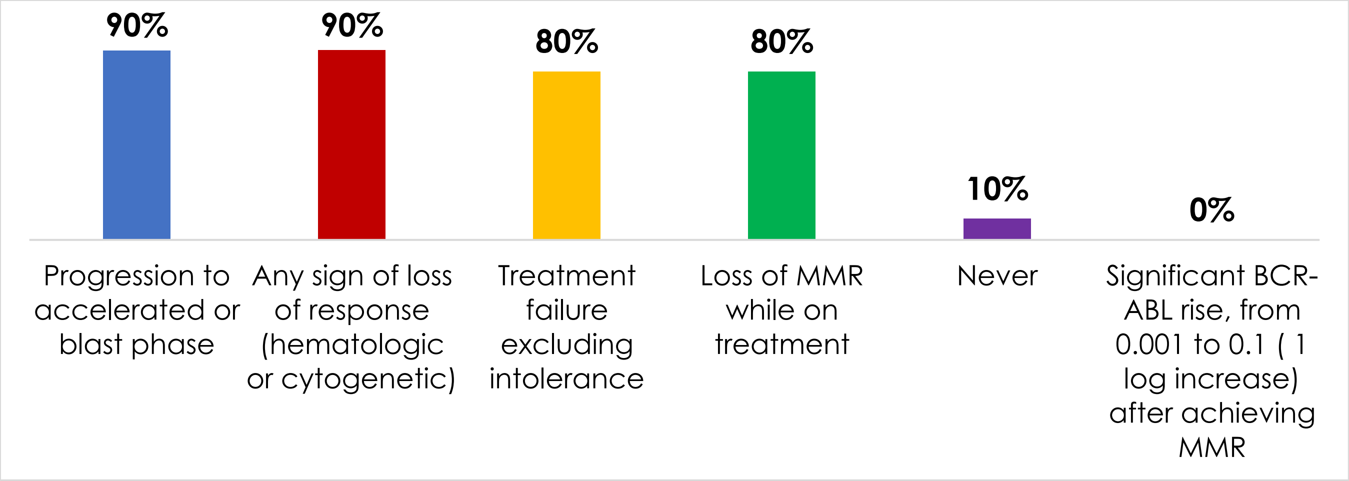

- **If "Never", why do you not assess mutation status?**

(Number of respondents= 1)

a) I don't find it useful
b) Financial issues
c) Lack of access

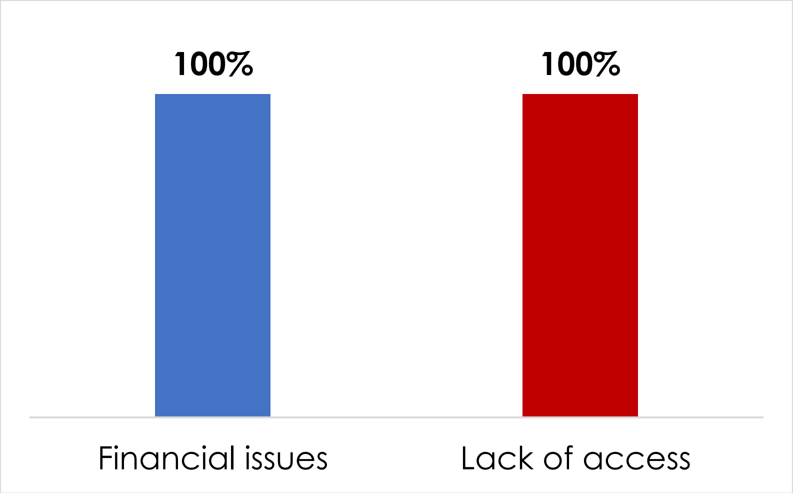

- **How frequently should the BCR-ABL be monitored during the first year of treatment and what is your practice?**

(Number of respondents=10)

Ideal:

a) Never
b) Every 3 months
c) Every 6 month
d) Once yearly

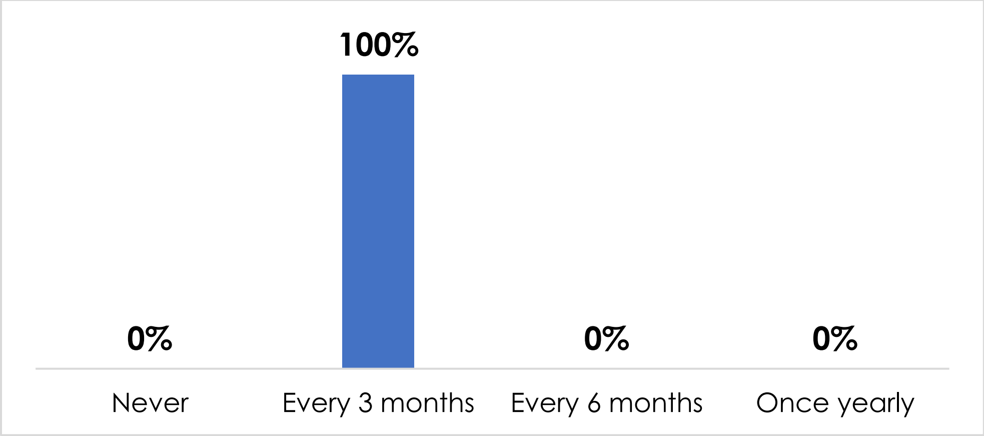

Current:

a) Never
b) Every 3 months
c) Every 6 months
d) Once yearly

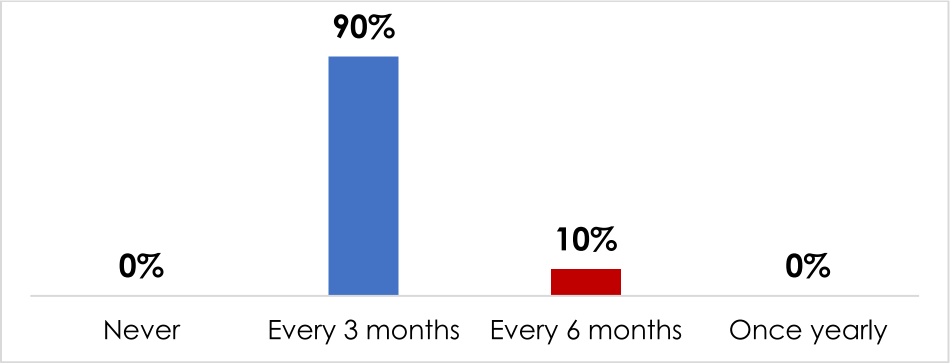

- **What is the main reason for not keeping to an ideal frequency, especially if the patient is followed by several physicians?**

(Number of respondents=13)

a) Cost of the test
b) Lab capability
c) Other

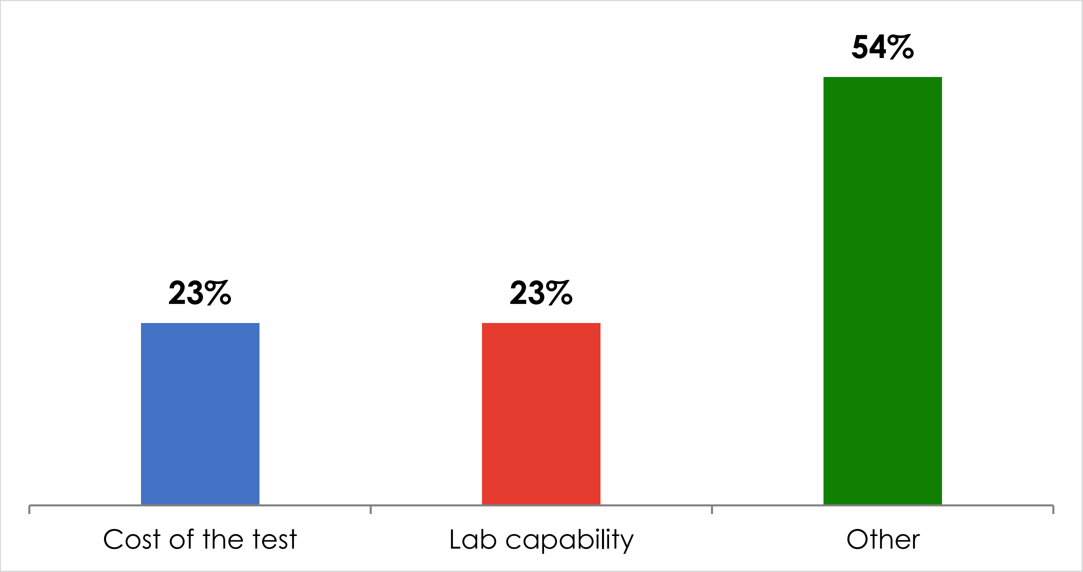

Treatment objectives

- **Please sort the following first-line therapy objectives in younger patients. Kindly rearrange them by dragging and dropping from 1 to 8, with (1) being the most important:**

(Number of respondents=10)

a) Achievement of EMR (less than 10% BCR-ABL at 3 months)
b) Overall survival
c) Achievement of MMR (major molecular response)
d) Prevention of progression to AP/BC
e) Achievement of CCR
f) Quality of life
g) Attempting treatment free remission
h) Achievement of DMR (at least MR4)
i) Minimize adverse events

1- Achievement of MMR (major molecular response)
2- Attempting treatment free remission
3- Achievement of DMR (at least MR4)
4- Overall survival
5- Achievement of EMR (less than 10% BCR-ABL at 3 months)
6- Prevention of progression to AP/BC
7- Achievement of CCR
8- Quality of life
9- Minimize adverse events

- **Please sort the following first-line therapy objectives in older patients. Kindly rearrange them by dragging and dropping from 1 to 8, with (1) being the most important:**

(Number of respondents=10)

a) Achievement of EMR (less than 10% BCR-ABL at 3 months)
b) Overall survival
c) Achievement of MMR (major molecular response)
d) Prevention of progression to AP/BC
e) Achievement of CCR
f) Quality of life
g) Attempting treatment free remission
h) Achievement of DMR (at least MR4)
i) Minimize adverse events

1- Overall survival
2- Prevention of progression to AP/BC
3- Achievement of MMR (major molecular response)
4- Quality of life
5- Achievement of CCR
6- Achievement of EMR (less than 10% BCR-ABL at 3 months)
7- Minimize adverse events
8- Achievement of DMR (at least MR4)
9- Attempting treatment free remission

- **What are your short-term treatment goals?**

(Number of respondents=10)

a) TFR
b) EMR followed by prolongation of overall survival
c) MMR

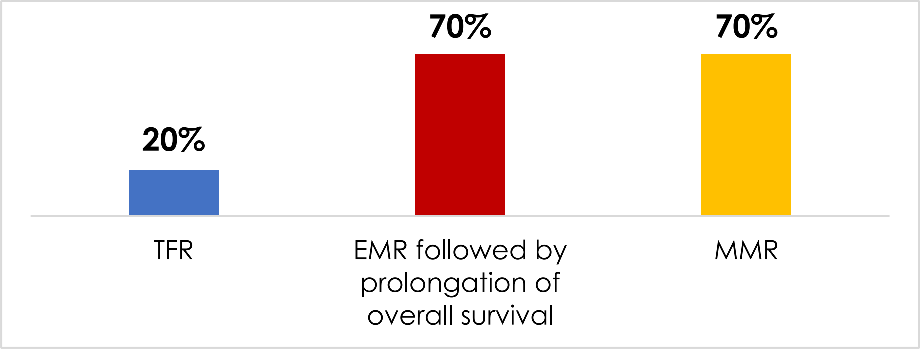

- **What are your long-term treatment goals?**

(Number of respondents=10)

a) TFR
b) EMR followed by prolongation of overall survival
c) MMR

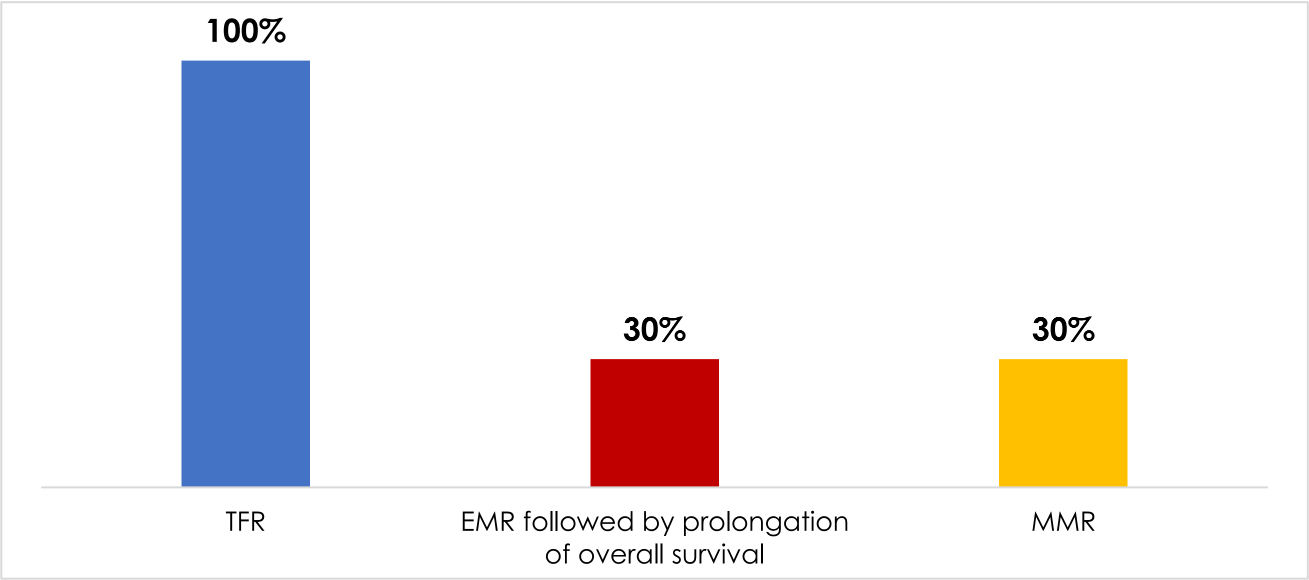

Treatment trends

- **In first line, please list the % of patients receiving:**

(Number of respondents=10)

a) Imatinib
b) Imatinib generic
c) Dasatinib
d) Nilotinib
e) Ponatinib
f) Bosutinib
g) Other treatment

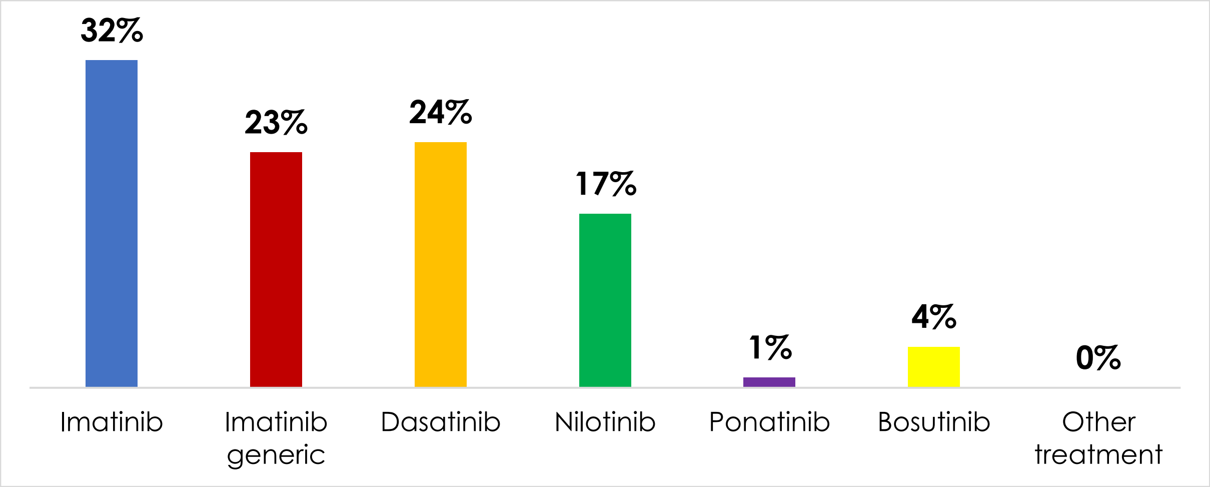

- **In second line, please list the % of patients receiving:**

(Number of respondents=10)

a) Imatinib
b) Imatinib generic
c) Dasatinib
d) Nilotinib
e) Ponatinib
f) Bosutinib
g) Other treatment

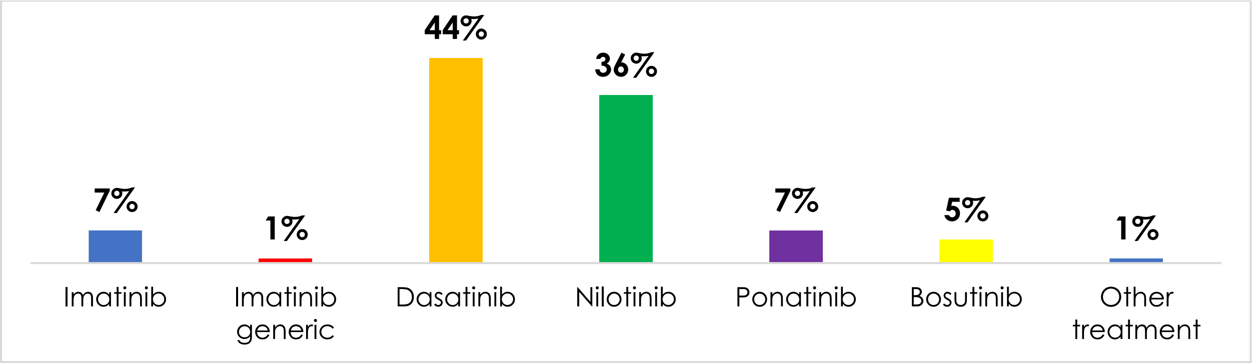

- **In third line, please list the % of patients receiving:**

(Number of respondents=10)

a) Imatinib
b) Imatinib generic
c) Dasatinib
d) Nilotinib
e) Ponatinib
f) Bosutinib
g) Other treatment

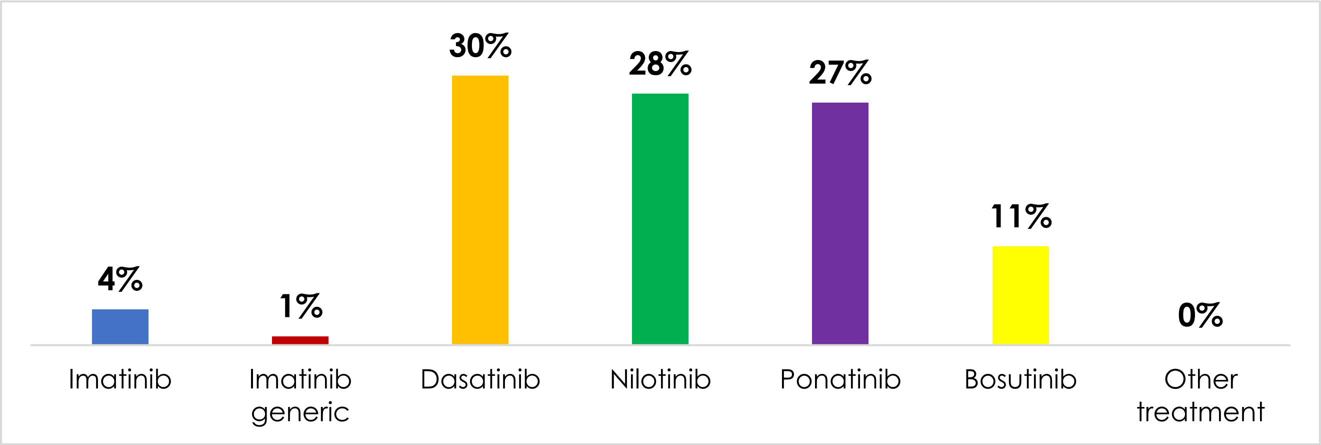

- **Please sort the following factors influencing your treatment decision for first-line therapy. Kindly rearrange them by dragging and dropping from 1 to 8, with (1) being the most important:**

(Number of respondents=10)

a) Sokal or EUTOS or ETLS score
b) Co-morbidities
c) Patient preference
d) Age
e) Dose regimen (QD/BID)
f) Treatment free remission attempt
g) Treatment availability

1- Sokal or EUTOS or ETLS score

2- Co-morbidities

3- Age

4- Patient preference

5- Treatment free remission attempt

6- Treatment availability

7- Dose regimen (QD/BID)

- **Under what circumstances would you select a second generation TKI over imatinib as frontline therapy for a patient with CP-CML?**

(Number of respondents=10)

a) When TFR is a high priority goal for the patient
b) For patients with high Sokal risk scores
c) For patients with low Sokal risk scores

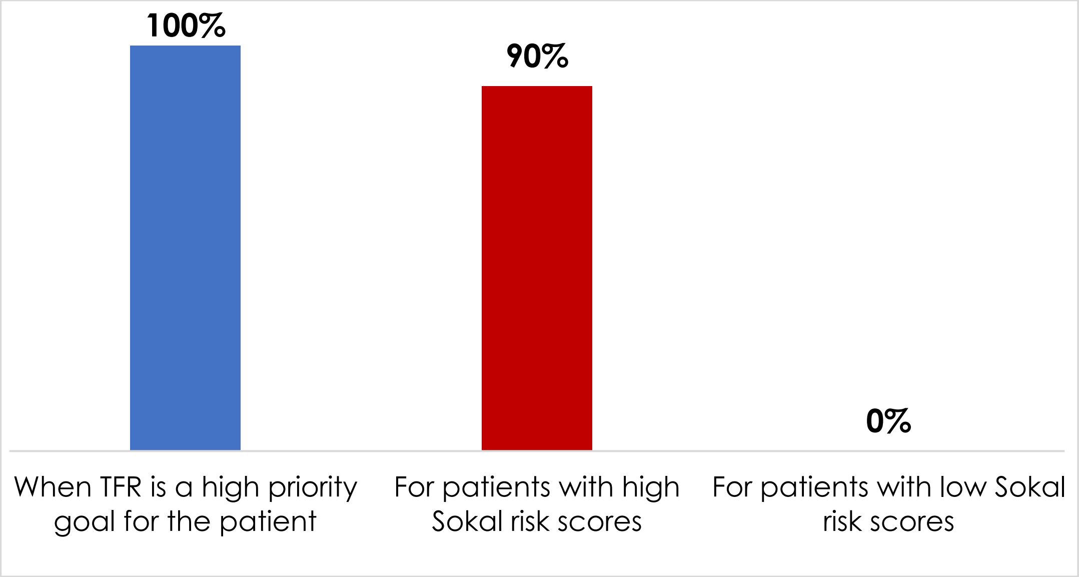

<u>**Treatment toxicities**</u>

- **For which kind of toxicities would you switch therapy?**

(Number of respondents=13)

Grade 3/4:

a) Hematological toxicity
b) Non-hematological toxicity

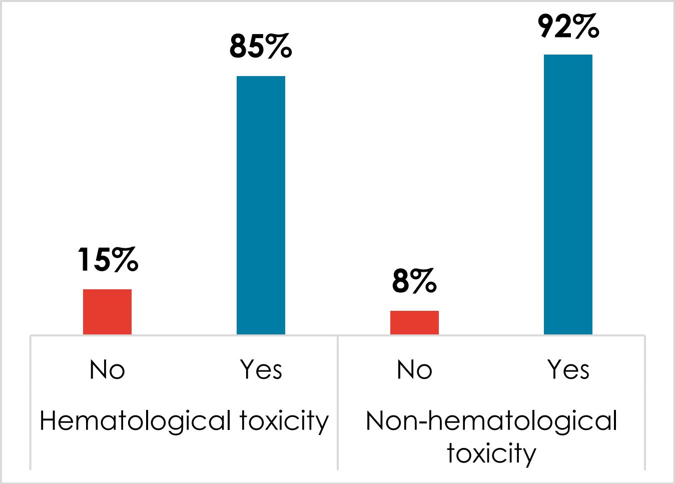

Persistent Grade 2:

a) Hematological toxicity
b) Non-hematological toxicity

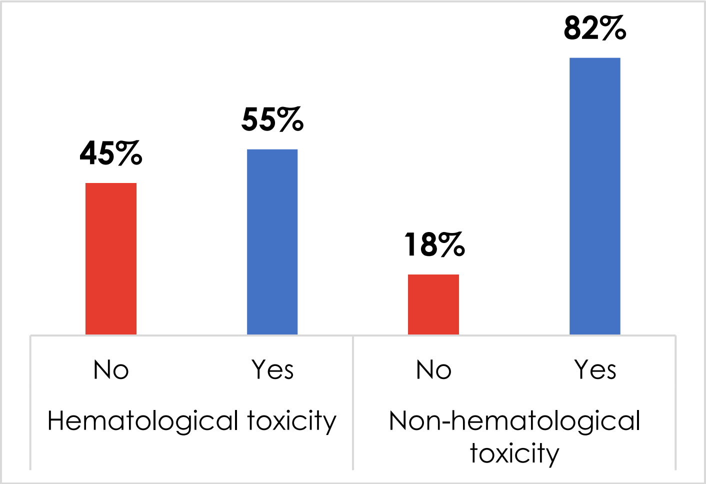

Persistent Grade 1:

a) Hematological toxicity
b) Non-hematological toxicity

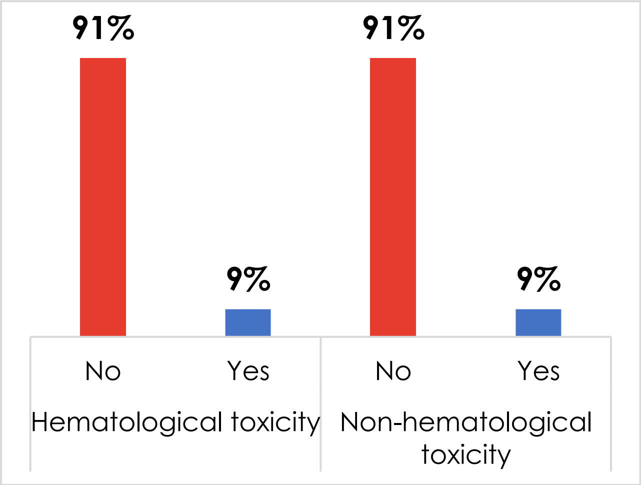

Treatment discontinuation

- **Are the below criteria well-met at your center?**

(Number of respondents=13)

a) Rapid intervention if the patient needs to restart therapy
b) Rapid turnaround of RQ-PCR results (within 4 weeks)
c) Capacity to test your patients every 4-6 weeks when required

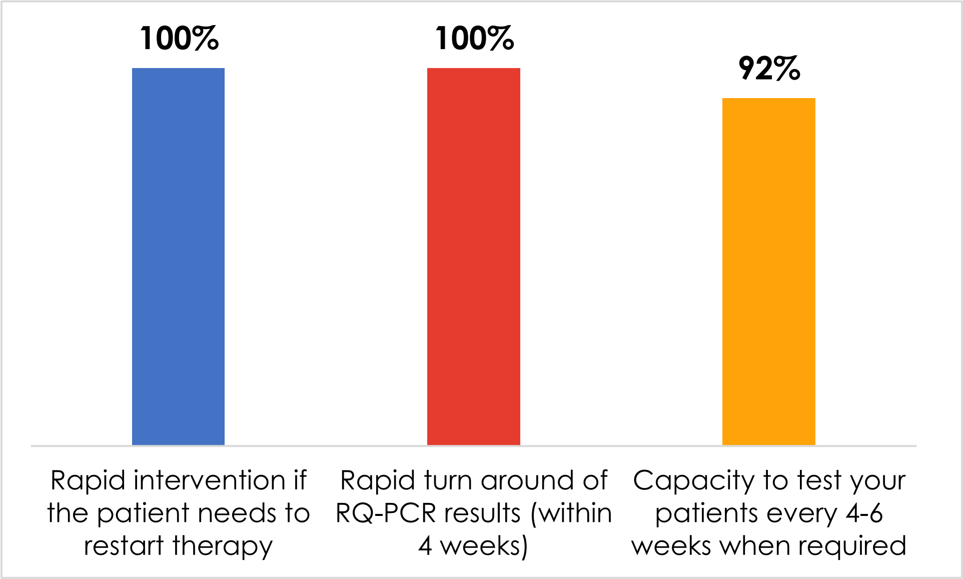

Supplement: Supplementary file 1 [file cancers-16-02114-s001.zip › cancers-2925873-supplementary.pdf]
